# The Effect of Lifestyle and Eating Habits on Obesity

**DOI:** 10.3390/nu17060993

**Published:** 2025-03-12

**Authors:** Alessandra Feraco, Mauro Lombardo, Andrea Armani

**Affiliations:** 1 Department for the Promotion of Human Science and Quality of Life, San Raffaele Open University, Via di Val Cannuta, 247, 00166 Rome, Italy; alessandra.feraco@uniroma5.it (A.F.); andrea.armani@uniroma5.it (A.A.); 2 Laboratory of Cardiovascular Endocrinology, San Raffaele Research Institute, IRCCS San Raffaele Roma, Via di Val Cannuta, 247, 00166 Rome, Italy

Obesity is a major global health problem and contributes to increased morbidity and mortality through its association with metabolic disorders, cardiovascular disease and other chronic pathological conditions. The complexity of obesity goes beyond energy imbalance and includes behavioural, dietary and physiological factors. This Special Issue of *Nutrients* explores the impact of lifestyle and dietary habits on obesity, providing new insights into dietary patterns, metabolic responses and behavioural adaptations that may influence weight management.

A significant contribution to this Special Issue was made by Suder et al., who studied the combined effects of aerobic–resistance exercise and an ad libitum high-protein, low-glycaemic-index diet on adipokine levels and dyslipidaemia in men with abdominal obesity. The results highlight the metabolic benefits of combining structured exercise with dietary modifications, leading to improvements in body composition and lipid profiles [1]. The role of dietary fibre in obesity and metabolic health was explored by Renall et al., who examined associations between fibre and adiposity and metabolic syndrome in Pacific European and New Zealand women. Their results show an inverse relationship between fibre intake and body fat percentage, reinforcing the importance of high-fibre diets in reducing the risk of metabolic disease [2]. An important question in obesity management is whether intermittent fasting offers advantages over continuous calorie restriction. Indeed, the efficacy of intermittent fasting in combating obesity and associated cardiovascular risk factors remains controversial. In their systematic review and meta-analysis, Siles-Guerrero et al. compare these two dietary approaches and conclude that although both lead to significant weight loss, fasting may offer additional short-term metabolic benefits, particularly with regard to insulin sensitivity. However, they emphasise the need for further research to determine long-term efficacy and adherence [3]. Food preferences and their relationship to food intake were analysed in a study by Nagai et al., who developed a Japan-specific food preference questionnaire to assess eating behaviour in subjects with abdominal obesity. Their results suggest that preferences for foods high in fat and carbohydrates are strongly correlated with actual food intake, highlighting the crucial role of food choices in the development of obesity [4]. Finally, Feraco et al. examined gender differences in the dietary patterns and eating behaviours of individuals with obesity, using principal component analysis (PCA) to classify different dietary profiles. Their results emphasise that structured eating behaviour, characterised by regular meal habits, no meal skipping, and eating in the company of others, is associated with a healthier body composition, whereas impulsive or irregular eating patterns may contribute to greater fat mass. These findings underline the need for gender-specific strategies in obesity management [5]. In summary, these studies highlight the multifactorial nature of obesity and the need to integrate dietary modifications, behavioural interventions and structured exercise programmes to achieve sustainable weight management [6]. Although specific dietary models, such as high-fibre diets and intermittent fasting, have demonstrated metabolic benefits [7], their long-term efficacy and individuals’ adherence remain key challenges [8]. The complexity of obesity requires a shift from general recommendations towards personalised nutritional interventions, supported by new findings on the impact of individual metabolic variability [9]. Recent studies have identified distinct metabolic phenotypes that influence responses to weight loss, suggesting that a more targeted approach could improve intervention outcomes [10].

Addressing obesity requires a paradigm shift beyond the simple energy balance model. Integrating findings from endocrinology, behavioural psychology and public health studies with parameters related to meal timing, food quality and gut microbiota composition enables the development of more effective nutritional plans tailored to the subject’s metabolic characteristics [11]. Notably, as a food group that must consistently be taken into account to maintain metabolic health, legumes have been proposed as a sustainable food to improve diet quality and support weight management due to their high fibre and protein content, which promotes satiety and improves glucose metabolism [12]. Chrononutrition has also emerged as a promising field, with evidence suggesting that aligning food intake with circadian rhythms can improve weight regulation and glycaemic control [13]. On the other hand, the role of ultra-processed foods in obesity continues to be debated, with recent analyses linking their consumption to increased adiposity and metabolic dysfunction, independent of caloric intake [14]. The moderate consumption of red wine has also been the subject of numerous studies for its possible beneficial effects on metabolic and cardiovascular health, although the debate remains open as to its real benefits in the context of a balanced diet [15].

Technological advances are also shaping the future of obesity management. Digital health tools, such as mobile apps and artificial intelligence-based diet coaching, have shown potential in improving adherence to lifestyle interventions [16]. Personalised behavioural interventions that incorporate machine learning algorithms to predict individual metabolic responses to dietary and exercise strategies may further improve success in weight loss and long-term maintenance [17]. The growing consensus in obesity research emphasises the need for interdisciplinary and evidence-based strategies that go beyond calorie restriction to address biological, behavioural and environmental determinants of weight regulation [18]. Future research should continue to refine personalised approaches, harnessing insights from emerging fields such as metabolomics, microbiome science and neuroendocrinology, to develop more sustainable and effective solutions for the prevention and treatment of obesity [19].

Overall, the studies featured in this Special Issue reinforce the idea that obesity is a multifaceted condition that requires an integrated, multidisciplinary approach. While diet modification, behavioural strategies and structured exercise programmes remain key factors in weight management, a growing body of research highlights the importance of tailoring interventions to individual metabolic, psychological and environmental characteristics. Advances in fields such as metabolomics, microbiome research and neuroendocrinology are shedding light on the biological heterogeneity of conditions that predispose individuals to obesity, emphasising the need for precision medicine in nutritional interventions. Ongoing research should aim to refine personalised approaches by incorporating technological innovations including artificial intelligence to optimise adherence and long-term success. By integrating this emerging knowledge into established lifestyle interventions, a more effective and sustainable framework for obesity prevention and treatment can be developed, ultimately improving metabolic health and reducing the burden of obesity-related diseases.
